# The clinician crowdsourcing challenge: using participatory design to seed implementation strategies

**DOI:** 10.1186/s13012-019-0914-2

**Published:** 2019-06-14

**Authors:** Rebecca E. Stewart, Nathaniel Williams, Y. Vivian Byeon, Alison Buttenheim, Sriram Sridharan, Kelly Zentgraf, David T. Jones, Katelin Hoskins, Molly Candon, Rinad S. Beidas

**Affiliations:** 1 Department of Psychiatry, University of Pennsylvania Perelman School of Medicine, Philadelphia, PA USA; 2 School of Social Work, Boise State University, Boise, USA; 3 Department of Medical Ethics and Health Policy, Perelman School of Medicine, University of Pennsylvania, Philadelphia, PA USA; 4 Leonard Davis Institute of Health Economics, University of Pennsylvania, Philadelphia, PA USA; 5 Center for Health Incentives and Behavioral Economics, Leonard Davis Institute of Health Economics, University of Pennsylvania, Philadelphia, PA USA; 6 Department of Family and Community Health, School of Nursing, University of Pennsylvania, Philadelphia, PA USA; 7 ideas42, New York, NY USA; 8 Department of Behavioral Health and Intellectual DisAbility Services, Philadelphia, PA USA

**Keywords:** Evidence-based practices, Participatory design, Implementation strategies, Community mental health

## Abstract

**Background:**

In healthcare settings, system and organization leaders often control the selection and design of implementation strategies even though frontline workers may have the most intimate understanding of the care delivery process, and factors that optimize and constrain evidence-based practice implementation within the local system. Innovation tournaments, a structured participatory design strategy to crowdsource ideas, are a promising approach to participatory design that may increase the effectiveness of implementation strategies by involving end users (i.e., clinicians). We utilized a system-wide innovation tournament to garner ideas from clinicians about how to enhance the use of evidence-based practices (EBPs) within a large public behavioral health system.

**Methods:**

Our innovation tournament occurred in three phases. First, we invited over 500 clinicians to share, through a web-based platform, their ideas regarding how their organizations could best support use of EBPs. Clinicians could rate and comment on ideas submitted by others. Second, submissions were judged by an expert panel (including behavioral scientists, system leaders, and payers) based on their rated enthusiasm for the idea. Third, we held a community-facing event during which the six clinicians who submitted winning ideas presented their strategies to 85 attendees representing a cross-section of clinicians and system and organizational leaders.

**Results:**

We had a high rate of participation (12.3%), more than double the average rate of previous tournaments conducted in other settings (5%). A total of 65 ideas were submitted by 55 participants representing 38 organizations. The most common categories of ideas pertained to training (42%), financing and compensation (26%), clinician support and preparation tools (22%), and EBP-focused supervision (17%). The expert panel and clinicians differed on their ratings of the ideas, highlighting value of seeking input from multiple stakeholder groups when developing implementation strategies.

**Conclusions:**

Innovation tournaments are a useful and feasible methodology for engaging end users, system leaders, and behavioral scientists through a structured approach to developing implementation strategies. The process and resultant strategies engendered significant enthusiasm and engagement from participants at all levels of a healthcare system. Research is needed to compare the effectiveness of strategies developed through innovation tournaments to strategies developed through design approaches.

## Background

A significant body of evidence supports the importance of evidence-based practice (EBP) in community mental health and health settings [1], yet these practices are largely underused by organizations and clinicians [2–4]. Implementation strategies are the interventions used to increase adoption, implementation, and sustainment of EBP in health services [5]. The evidence base for implementation strategies has advanced significantly in recent years including the identification of a complex set of factors related to implementation success. These factors include organizational- and individual-level factors, as well as characteristics related to the intervention, and the economic, political, and social context [6–8]. Although there are several proposed typologies of implementation strategies [5], no systematic approach to developing these strategies has been proposed [9]. Moreover, implementation strategy development has rarely systematically incorporated stakeholder involvement in the design [10], a critical oversight given research in healthcare suggesting that stakeholder input results in more effective outcomes [11, 12]. These weaknesses may explain why many implementation strategies fail to improve either implementation or clinical outcomes and why most strategies fail to engage their targeted mechanisms of action [13].

The importance of participatory approaches has been long recognized [14]. Multiple research traditions and disciplines such as implementation science, community-partnered research, and action research have incorporated provider input through stakeholder engagement methods and dynamic partnership [14–18]. Participatory design approaches are lauded for creating effective implementation strategies because they provide systematic methods to include and empower stakeholders at the beginning stages [19–21], and provide opportunities for stakeholders to be involved in design [14]. The current study utilizes an innovation tournament, a structured participatory design approach, in community mental health, as a springboard for implementation strategy co-design. Innovation tournaments are a form of crowdsourcing in which a host issues a call for ideas to address a specific challenge or problem within a system, and frontline providers and staff who work within the system are invited to submit their ideas for how to address the challenge. After multiple steps of screening and evaluation, a few ideas emerge as winners and others do not advance. Systematic review evidence points to the effectiveness of such crowdsourcing participatory design strategies in generating novel and useful solutions to complex and intractable problems in areas outside of healthcare settings [22–24].

The aims of the current study were twofold: (a) to test the utility of an innovation tournament for developing participatory-informed implementation strategies that enhance the use of evidence-based practices (EBPs) within a large behavioral health system and (b) to generate ideas from clinicians about the best ways for organizations to facilitate EBP implementation. Our study adds to the literature in several ways. First, our study extends the current literature on participatory approaches in implementation science, specifically providing proof-of-concept of the utility of the innovation tournament approach. We are aware of only one other study that applied the innovation tournament methodology to problems in healthcare systems and that study included a single organization focused on improving patients’ experience of care [24]. Second, we extend this work by using an innovation tournament to address EBP implementation and by applying the method across an entire behavioral health system comprised of a network of 200 independent treatment organizations (of mental health and substance use disorders) which are affiliated only to the extent that they are subject to the same policy and funding constraints through a single policy and payer infrastructure. Third, we collected ideas directly from the stakeholders who will be targeted by implementation strategies and have the most intimate understanding of factors that could affect the implementation process. The innovation tournament provided a structured and efficient way to integrate insights from clinicians (who have unique expertise about the problem), system leaders (who can speak to feasibility and financing), and experts in behavioral economics who understand drivers of implementation-relevant behavior. Behavioral science, including psychology and behavioral economics, can contribute valuable information about the best way to structure implementation efforts to optimize the design of implementation strategies that target organizations, critically important given the scarcity of resources in the mental health system.

Due to past successful experiences with recruitment within this system, we anticipated high engagement and a successful tournament. We had one hypothesis regarding the content of ideas generated in the tournament: In our past work, financial challenges such as the scarcity of resources in the mental health system and the organizational financial investment required to support EBPs have been highlighted by all stakeholders [6, 25]. In addition, clinicians have previously reported that they do not feel incentivized by their provider organizations to implement EBPs [26]. We hypothesized therefore that the majority of ideas offered by clinicians would be related to compensation and financial incentives.

## Method

This study was approved by the Institutional Review Boards of the University of Pennsylvania and City of Philadelphia.

### Setting

The Department of Behavioral Health and Intellectual disAbility Services (DBHIDS) in Philadelphia is a large publicly funded behavioral health system that annually services approximately 169,000 individuals with mental health and substance use disorders. Since 2007, DBHIDS has supported the implementation of multiple EBPs [6, 26, 27] including cognitive behavior therapy [28, 29], trauma-focused cognitive-behavioral therapy [30], prolonged exposure [31], dialectical behavior therapy [32], and parent-child interaction therapy [33]. DBHIDS funds training and consultation in line with treatment developer recommendations, and staff time to coordinate implementation, training, and ongoing consultation by treatment developers for each of the five EBPs. In 2013, the Evidence-Based Practice and Innovation Center (EPIC) was launched as a centralized infrastructure for EBP administration. In addition to supporting the EBP initiatives, which predated the creation of this entity, EPIC aligned policy, fiscal, and operational approaches to EBP implementation by developing systematic processes to contract for EBP delivery, hosting events to publicize EBP delivery, designating providers as EBP agencies, and creating enhanced rates for the delivery of some EBPs [27].

### Procedure

This innovation tournament procedure was borrowed from Terwiesch and colleagues [24], developed through the Penn Medicine Center for Health Care Innovation [34].

#### Recruitment

We invited, by email, publicly funded behavioral health organization leaders (*n* = 210), clinicians (*n* = 527), and other community stakeholders (e.g., leaders of community provider organizations, directors of a clinician training organization; *n* = 6) in Philadelphia to participate in an innovation tournament called “The Philly Clinician Crowdsourcing Challenge.” We also e-mailed the invitation to four local electronic mailing lists known to reach large swaths of the network (e.g., the managed care organization listserv, the community provider organization listserv). Leaders and stakeholders were asked to forward the email to clinicians, so it is impossible to know exactly how many clinicians received and read the request. Moreover, some clinicians may have received the invitation more than once (i.e., from a direct e-mail and from their executive director). We sent leaders and stakeholders a priming email 1 week prior to the start of the tournament [35]. We then sent an initial invitation e-mail containing the link to participate and four reminder emails over the course of 5 weeks. The innovation tournament link was live for 5 weeks from February to March 2018. Potential participants clicked on the link and provided consent.

#### Tournament platform

The “Your Big Idea Platform,” powered by the Penn Medicine Center for Health Care Innovation, is a web-based platform that enables crowdsourcing for ideas and solutions. Idea challenges are posted to the platform, and participants can submit an idea, rate other ideas on a 1–5 “star” scale, and comment and rate ideas submitted by other participants [24].

#### Idea elicitation prompt

The tournament prompt (i.e., the question posed to potential participants) was developed through an iterative process. The authors created an initial draft of the tournament prompt and solicited feedback from the project’s steering committee. Our team conducted four cognitive interviews with clinicians and policy-makers to receive feedback on the wording, score, and design of the prompt. The final prompt asked clinicians, “How can your organization help you use evidence-based practices in your work?” Participants could submit and rate as many ideas as they wished.

#### Idea vetting and selection of winners

We assembled an expert panel of stakeholders to vote on the ideas and determine the winners. We wanted the committee to represent the perspectives of those who (a) understand what is feasible and acceptable to clinicians, (b) are in a position to implement the ideas, and (c) can evaluate the idea’s potential to leverage principles of behavioral economics. Therefore, the Challenge Committee included two agency stakeholders, two city administrators (including the Commissioner of the Philadelphia Department of Behavioral Health and Intellectual DisAbility Services [DBHIDS]), and two behavioral science experts. To minimize burden on our Challenge Committee, the research team refined the list from 65 to 22 by eliminating ideas that were not actionable (e.g., increase inpatient stays to a minimum of 60 days) or too similar to another, better-elaborated idea. The committee was asked to rate the ideas based on their enthusiasm for the idea’s “potential to increase therapist use of evidence-based practice” using a 1–5 Likert scale (1 = not at all enthusiastic, 5 = very enthusiastic). The top six ideas as rated by the Challenge Committee were chosen as winners.

Participants who submitted winning ideas (hereafter referred to as “innovators”) were invited to a working lunch with our team to refine and develop their idea leveraging principles of behavioral economics. For example, innovators discussed how to amplify the impact of their proposed strategy using such behavioral economic principles as changing defaults, peer comparisons, loss-framed incentives, and nudges [36–39].

During this meeting, innovators prepared a brief presentation summarizing their idea for a community-facing event, *The Idea Gala*, held in May 2018. All who received the invitation to participate in the tournament (described above) were invited to the event. During The Idea Gala, each of the six innovators presented their idea for 3 min. Attendees had the option of using an online polling system (www.mentimeter.com) to answer the following question on a 1–10 Likert scale (1 = not at all excited, 10 = very excited): “How excited are you by this idea?” and to submit brief comments about the ideas. After the presentation of each idea, there were 10 min of questions from the audience and an unstructured discussion of the idea by the Challenge Committee. Challenge Committee members focused their comments on the problem, the solution, applications of principles of behavioral economics to enhance implementations, metrics for success, and resources necessary for implementation.

#### Coding and categorization of ideas

After reviewing submitted ideas, we developed a coding scheme to categorize the ideas into eight non-mutually exclusive categories. These categories included (a) training, (b) financing and compensation, (c) clinician support and preparation tools, (d) client support, (e) EBP-focused supervision, (f) changes to the scope or definition of EBPs, (g) changes to the system and structure of publicly funded behavioral health care, and (h) other. Two study team members (RS and VB) categorized all the ideas, and inter-rater reliability was found to be excellent (*K* = .92). Discrepancies were resolved through discussion.

## Results

### Tournament feasibility and respondent characteristics

A total of 65 ideas were submitted by 55 participants. The participants represented 38 behavioral health organizations representing multiple levels of care (e.g., outpatient, inpatient, residential) and specialties (mental health, substance use). The majority of participants were white (61.8%) and female (61.8%). Additionally, 59 participants submitted 899 ratings, and 22 participants submitted 55 comments on the 65 ideas. Of the clinicians (*n* = 527) who were contacted directly to participate in the tournament, 12.3% submitted an idea. According to personal communications with “Your Big Idea” staff, this response rate is consistent with (or exceeds) the average response rate (5%) of challenges administered through the “Your Big Idea” platform.

### Categories of solutions

Submitted ideas encompassed all aspects of supporting EBPs. We categorized ideas into eight categories. Over 40% of ideas pertained to training (42%), followed by ideas related to changes to financing and compensation (26%), including paid training, financial incentives, and rewards. A large proportion of ideas included clinician support and preparation tools (22%) and supervision (17%). Other categories included supporting clients (5%), changes to the scope or definition of EBPs (8%), and changes to the system and structure of publicly funded behavioral health care (6%). See Table 1 for examples of ideas and ratings by category by other participants.

**Table 1:** Categories of solutions

Category^a^	Example ideas	Tournament ratings^b^
Training (*n* = 27; 42%)	• Training delivered by EBP trainer/expert on-site at the organization to train all clinicians • EBP training for managers and supervisors • Shorter and more frequent training sessions	4.03 (1.08) *n* = 396
Financing and compensation (*n* = 17; 26%)	• Paid training, workshops, and supervision • Enhanced rate for clinicians who report EBP use in progress notes • Incremental bonus program for committing to stay at organization	4.13 (1.08) *n* = 278
Clinician support and preparation tools (*n* = 14; 22%)	• Yoga classes for clinicians provided by organization • Collecting data on client outcomes to inform treatment • Binder with EBP “one-pagers” to guide treatment by session	3.76 (1.18) *n* = 196
Supervision (*n* = 11; 17%)	• Role-play using EBP in supervision • Access to designated EBP experts when supervisors at organization are not familiar with EBP • Group supervision with walk through of identifying to use EBP with clients, how to use it, and how to address barriers to using EBP	4.03 (1.13) *n* = 202
Other (*n* = 7; 11%)	• Support for family-involved treatments • Partner with local universities to provide organizations with master’s level interns with EBP knowledge and experience • Measure client satisfaction with EBP	3.56 (1.45) *n* = 71
Change to the scope or definition of EBPs (*n* = 5; 8%)	• Change EBPs to integrate family, community, and social determinants • Develop trans-inclusive EBPs	3.85 (1.17) *n* = 78
Change to the system and structure of publicly funded behavioral health care (*n* = 4; 6%)	• Mandatory use of EBPs • Hiring staff for case management • Fewer clients and longer sessions in non-profit organizations	3.86 (1.24) *n* = 70
Client support (*n* = 3; 5%)	• Pre-session worksheets to prepare client for session • On-site childcare provided by organization • Welcoming and calm office spaces	3.67 (1.30) *n* = 30

^a^Sixteen ideas were categorized in two or more categories, so the total percentages add to over 100% and the total number of ratings add up to over 899

^b^Clinicians were invited to participate in the innovation tournament by rating submitted ideas. Clinicians were asked to rate submitted ideas on a scale of 1–5 stars. The average and standard deviations of the ratings are presented in this column

### Winning ideas and The Idea Gala

See Table 2 for the Challenge Committee’s ratings of the top winning ideas, each innovator’s idea, and the target problem addressed by the idea.

**Table 2:** Top six innovator ideas

Title	Idea	Issues addressed through idea	Challenge Committee rating ranking (*n* = 6)^a^	GALA attendee rating ranking^b^
Pre-session preparation	Create a relaxing waiting room that helps prepare the patient (along with a “pre-session quick sheet”) for the session ahead	Stress created from chaotic waiting room that detracts from session time and opportunities to implement EBPs	1	1
Experts in your back pocket	Network of EBP experts are available for consultation and supervision via a hotline or face-to-face appointments	Lack of experts on various EBPs within organizations	2	5
Workshops and rewards	Point-based system for EBP fidelity and workshop attendance that can be used towards session supplies or gift cards	Clinician burnout and lack of incentive for clinicians	3	4
Electronic evidence-based screening instrument inventory	Provide validated screening instruments in the EMR to clinicians to easily assess treatment needs and track client progress	Barriers to implementing measurement-based care	4	6
On-site training and fidelity follow up	A certified EBP trainer comes to an organization and provides on-site training and consultation	Lack of EBP support within organization and barriers to attending distant training sessions	5	2
Community-based mentoring program for EBP	Implement an intra-agency EBP mentoring program that addresses therapist isolation and lack of community understanding from EBP experts	Therapist isolation and lack of community understanding from EBP experts	6	3

^a^Challenge Committee members were asked to rate all the ideas based on their enthusiasm for the idea’s “potential to increase therapist use of evidence-based practice” using a 1–5 Likert scale. The top 6 ideas (presented here) were chosen, and the ranking of the ideas are presented in this column

^b^GALA attendees were asked “How excited are you about the idea” following the presentations at the IDEA GALA and ranked ideas using a 1–10 Likert scale. The ranking of the top 6 ideas are presented in this column

A total of 85 participants (12 project staff, 6 Challenge Committee members, 6 innovators, 61 other attendees) representing 22 organizations attended The Idea Gala. Attendees’ ratings of the innovators’ ideas are shown in column 3 of Table 2, alongside the Challenge Committee’s ratings in column 2. Both groups rated “pre-session preparation,” defined as creating a relaxing waiting room to prepare clients for the upcoming session, the highest among the 6 winning ideas.

## Discussion

The present study is the first to utilize an innovation tournament to crowdsource ideas from community clinicians about how they can best be supported to use EBPs. The main purpose and benefit of an innovation tournament is to engage a community through participatory design to provide their best ideas. While many studies have included stakeholder elicitation of suggestions, preferences, and targets for intervention (e.g., [40]), an innovation tournament has a unique contribution as a participatory method for the following reasons. First, an innovation tournament can engage an infinite number of people in a fun and creative way for low incremental cost, while giving each participant an equal voice with the submission of an idea. A tournament allows for quick identification of potentially high impact ideas at every level of a system and can provide a training ground to empower stakeholders’ good ideas and improve their ability to express them. Lastly, a tournament provides public acknowledgement of people who come up with ideas, and this may increase buy-in to the ideas when ultimately implemented (the “Ikea effect”) and encourage future participation. Innovation tournaments are therefore a relatively inexpensive and efficient way to solicit and powerfully engage end-user input.

Our tournament was a success in this regard: we had 65 ideas submitted by 55 participants and 899 ratings of ideas by those participants. The number of submissions and overall participation exceeded our expectations, and we engaged many more in our community through our celebratory event. The study team was continuously struck by the high level of community stakeholder enthusiasm throughout the study period. While use of crowdsourcing in health research is nascent [22], this study demonstrates how an innovation tournament can be not only feasible to implement across an entire system, but also a successful and acceptable means to engage a community of service providers in sharing their expertise to generate useful implementation strategies that address local barriers, contexts, and populations.

The prompt for our innovation tournament was purposefully broad in order to encourage clinicians to share their unique local knowledge about targeted barriers that interfere with EBP implementation and their innovative ideas for overcoming those barriers. We wanted to capture the full spectrum of levers that support clinicians in implementing EBPs [34]. The most common theme reflected in the ideas involved training. This was surprising given that over the last 10 years, DBHIDS has supported implementation of several EBPs in over 50 mental health and substance use provider agencies by funding training, consultation, coordination, administration through EPIC, and in some cases reimbursing for lost therapist time, in addition to providing an enhanced reimbursement rate [26, 27]. Despite this, clinicians within this system overwhelmingly reported that additional (free) training would increase their use of EBPs with their clients. Obviously, active training is an important vehicle to change therapist behavior; training also impacts knowledge and attitudes [41]. Nonetheless, training is not the answer to every implementation problem: even clinicians trained via current gold standard approaches (i.e., workshop, manual, and clinical supervision) often do not demonstrate fidelity [42].

One alternative and more parsimonious explanation for well-trained clinicians’ thirst for additional training is that, put simply, EBPs are hard. Almost every EBP requires a skilled interventionist to deliver a complex, multi-component repertoire of behaviors, while responding to the client’s inputs and needs. It is striking that two thirds of the ideas put forth in the tournament target training and/or clinician support tools. There is also evidence from the research literature suggesting that trained clinicians do not feel confident delivering EBPs [43]. If we start with an assumption that clinicians want to deliver EBPs and would like to feel confident that they can enact effective treatment, we might accelerate implementation if we invest in implementation strategies that make our treatments easier to do, rather than additional training initiatives. Future research on implementation strategy design might draw on insights from behavioral economics such as “nudges” and changing the choice architecture; simple checklists can make multistep procedures easier [44–46].

Consistent with our hypothesis that a significant portion of ideas would be financially motivated, over a quarter of the submitted ideas pertained to compensation and pay. As we found in our prior research, stakeholders note that EBPs are associated with higher marginal costs that need to be reimbursed and that existing reimbursement strategies rarely cover these higher costs [6, 25]. While there were ideas calling for additional lump compensation, there were relatively few ideas involving complex incentive structures such as those used in pay-for-performance or value-based payment models. This may reflect the “bottom up” approach of the innovation tournament; involving clinicians helped us identify new opportunities that would be overlooked if innovation was left to administrators and executives alone. Ultimately, a combination of “top down” and “bottom up” approaches may tell us most about how to motivate complex, expensive repertoires of behavior such as those required to implement EBPs.

There are limitations to the study design and methodology that deserve mention and highlight avenues for future research. First, because it draws from one system that is clearly aligned with promoting EBPs—this sample may not be representative of clinicians in this system or other public behavioral health systems. The specificity of implementation strategies that emerge from an innovation tournament conducted within a given service system is both an advantage and a limitation of this approach—the strategies developed through the tournament may be ideal for a particular context and system but may not generalize to other contexts and systems. Second, beyond “winning” the tournament, clinicians in our study were not promised that their ideas would be implemented. This may have restricted clinicians’ willingness to participate or encouraged ideas that were not “implementable.” Given evidence suggesting discordance between behavior and stated preferences [47], we framed tournament ideas as an “input” to design rather than relying entirely on stated preferences. Moving these inputs to design will require additional scholarship and analysis. Currently, we are re-analyzing the ideas emphasizing behavioral processes and barriers, not just elicited preferences, as part of a multifaceted approach towards implementation strategy design. Third, although our response rate was more than double that of the average innovation tournament, some tournaments have generated much higher participation rates, even in larger organizations. Future research should examine how participants’ expectations regarding the eventual implementation of their ideas influence engagement. Fourth, we elected to form a Challenge Committee of city administrators, agency stakeholders, and experts in behavioral economics to decide on the winners; a challenge committee comprised of different stakeholder groups (e.g., clinicians, patients) may have selected different strategies as winners. Future studies should compare whether different stakeholder groups select the same or different strategies and, most importantly, whether these choices predict the effectiveness of strategies used to improvement EBP implementation within the system.

## Conclusion

Through a novel methodology for participatory design, findings from this study highlight the feasibility and utility of engaging clinicians—arguably the ultimate target of implementation strategies—through a structured, system-wide innovation tournament. Moreover, the innovation process does not have to end with the final and winning ideas [24]. Analysis of all of the ideas (or better yet, re-engagement with those who did not have winning ideas) can provide a fertile ground for future research. We believe the tournament succeeded in engaging stakeholders with critical expertise and provided valuable data that furthers the science of developing implementation strategies to improve the implementation of EBPs. More importantly, the success of our tournament was in engaging and empowering the community. Although we did not measure acceptability directly, the overwhelming enthusiasm for this project from clinicians, agency administrators, and city officials indicated to us that clinicians were pleased to be queried and felt validated that their ideas and viewpoints were not only heard, but celebrated. The clear implication to us is that involving clinicians in every stage of implementation (from strategy design to sustainability) consistent with community-participatory research is essential to designing effective implementation strategies that improve the quality of community-based care.

## Data Availability

Data will be made available upon request. Requests for access to the data can be sent in the Penn ALACRITY Data Sharing Committee. This Committee is comprised of the following individuals: Rinad Beidas, PhD, David Mandell, ScD, Kevin Volpp, MD, PhD, Alison Buttenheim, PhD, MBA, Steven Marcus, PhD, and Nathaniel Williams, PhD. Requests can be sent to the Committee’s coordinator, Kelly Zentgraf at zentgraf@upenn.edu, 3535 Market Street, 3rd Floor, Philadelphia, PA 19107, 215-746-6038.

## Abbreviations

DBHIDS: Department of Behavioral Health and Intellectual disAbility Services (DBHIDS)
EBP: Evidence-based practice
EPIC: Evidence-Based Practice and Innovation Center
